# In memoriam: Jacques E. Dumont (1931-2023)

**DOI:** 10.3389/fendo.2023.1171677

**Published:** 2023-03-16

**Authors:** Guy G. Rousseau, Pierre De Meyts

**Affiliations:** De Duve Institute, Catholic University of Louvain, Brussels, Belgium

**Keywords:** obituary, Jacques E. Dumont, ULB, thyroidology, Mont Ste Odile Symposium

A giant figure of European endocrinology has left us. Jacques Émile Dumont, emeritus professor at the Free University of Brussels (ULB), passed away on February 6, 2023. His prime interest focused on the pathophysiology of the thyroid gland, extended to the wider issues of receptor signal transduction, molecular genetics and cancer. He also had a tremendous understanding of theoretical and systems biology. With his ULB team and long-time associate Gilbert Vassart, he made major contributions to these fields, publishing more than 500 scientific papers.

Jacques graduated as M.D. from the ULB in 1956 and, after a NIH fellowship with John B. Stanbury at the Massachusetts General Hospital in Boston (1958-1959) and a residency in Internal Medicine at the University Hospital St Pierre, he obtained in 1965 a Belgian certification in Clinical Biochemistry and a Ph.D. degree in Biochemistry from the ULB School of Medicine where he was appointed professor in 1971. In the meantime, he became an established investigator of the Belgian Scientific Research Fund (FNRS). He was visiting professor at the Vrije Universiteit Brussel and at the Graduate School of Biomedical Sciences, University of Texas (Houston).

Jacques was a renowned scientist of many trades, all devoted to the progress, teaching and applications of medical research. As a physician, Jacques kept seeing patients in clinical endocrinology throughout his career, helping him to make important clinical discoveries: The definition of endemic myxoedematous cretinism and of non-autoimmune hereditary hyperthyroidism as distinct diseases; the identification of somatic gain-of-function mutations of the TSH receptor as a cause of hyperfunctioning thyroid adenoma, loss-of-function mutations of this receptor as one cause of congenital hypothyroidism, and a mutation altering the recognition specificity of a GPCR in hCG-dependent hyperthyroidism during pregnancy. This led to generation of the first transgenic models of thyroid tumorigenesis, of diagnostic tools for congenital hypothyroidism due to defects in thyroid-specific genes, and of assays for TSH function and for thyroid stimulatory antibodies causing Graves disease.

As a basic science investigator, Jacques provided the background of these advances. The contributions of his team to fundamental research include the identification of cyclic AMP as a mitogenic and tumorigenic agent in some tissues, an unexpected property that explains thyroid adenoma and congenital hyperthyroidism; the mechanism of the control of the thyroid gland by iodide; the structure of thyroglobulin; the use of ‘low-stringency’ PCR to clone a number of GPCRs, including the TSH receptor; a molecular model of glycoprotein hormone receptors activation and its negative cooperativity; the role of negative feedback suppression in tumorigenesis.

Jacques was always eager to promote collaboration in medical research in order to both foster scientific progress and muster financial resources. One way was to initiate national and international research projects. Another was to gather a critical mass of investigators on the Erasmus campus of the ULB hospital in Brussels: he founded the Institute of Interdisciplinary Research in Human and Molecular (formerly Nuclear) Biology (IRIBHM) in 1968 and was the Institute’s director until 2001. Afterwards, Jacques remained dedicated to his brainchild by maintaining an active presence in his laboratory until the very last year of his life. His concern for increasing financial support of young investigators materialized in setting up, with Prof. Pierre Lekeux (University of Liège) and one of us (G.G.R.), the WelBio program (Walloon Excellence in Lifesciences & Biotechnology) in 2009. Jacques was also an entrepreneur. He founded several start-up companies at a time when this was still considered a departure from standard academic policies.

Another of his achievements was the organization and presiding of scientific meetings: NATO courses on Cyclic Nucleotides (1974) and on Cell Regulation by Intracellular Signals (1980), the 4th International Conference on Cyclic Nucleotides in Brussels (1980), the 1981 European Thyroid Association (ETA) meeting in Brussels, the European Group on Irradiation and Thyroid Diseases (1982, 1984) and the 2003 FEBS meeting in Brussels. Most prominent was the popular and successful yearly European Symposium on Hormones and Cell Regulation in Mont Sainte Odile (Ottrott, Alsace) which Jacques founded in 1976 and presided until his death. His aim was to strengthen European scientific relationships, and in particular Franco-German reconciliation, hence the symbolic choice of the venue on the border between the two former opponents. The 46^th^ edition of this symposium will take place on September 6-9, 2023, organized by David Carling and Maria Sibilia, with Bernard Payrastre now at the helm of the scientific committee. An annual Jacques Dumont lecture was established from 2018, and the Jacques Dumont prize will be awarded to a talented young scientist attending the meeting.

**Figure 1:**
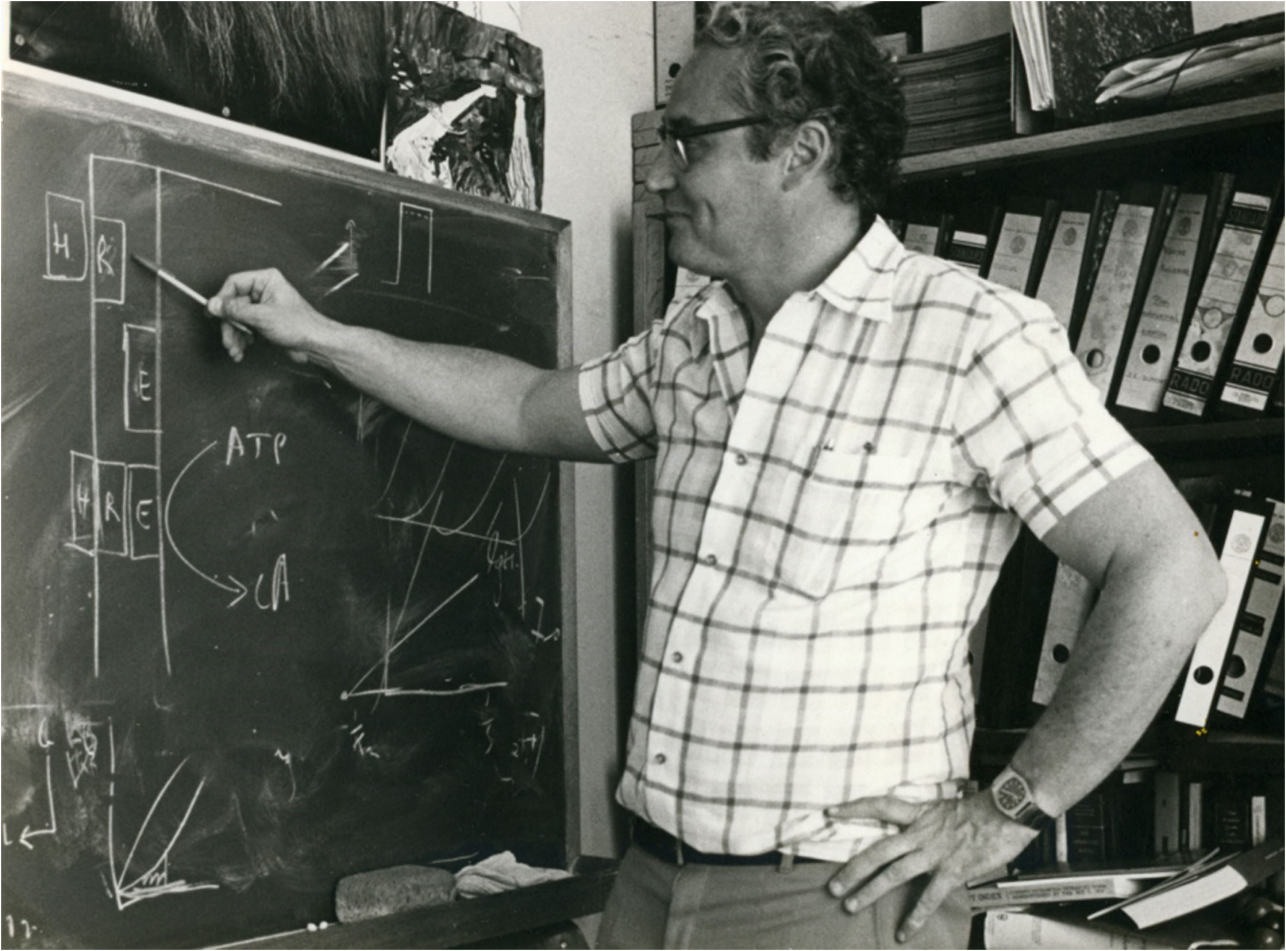
The early days of Jacques' receptor theory.

**Figure 2:**
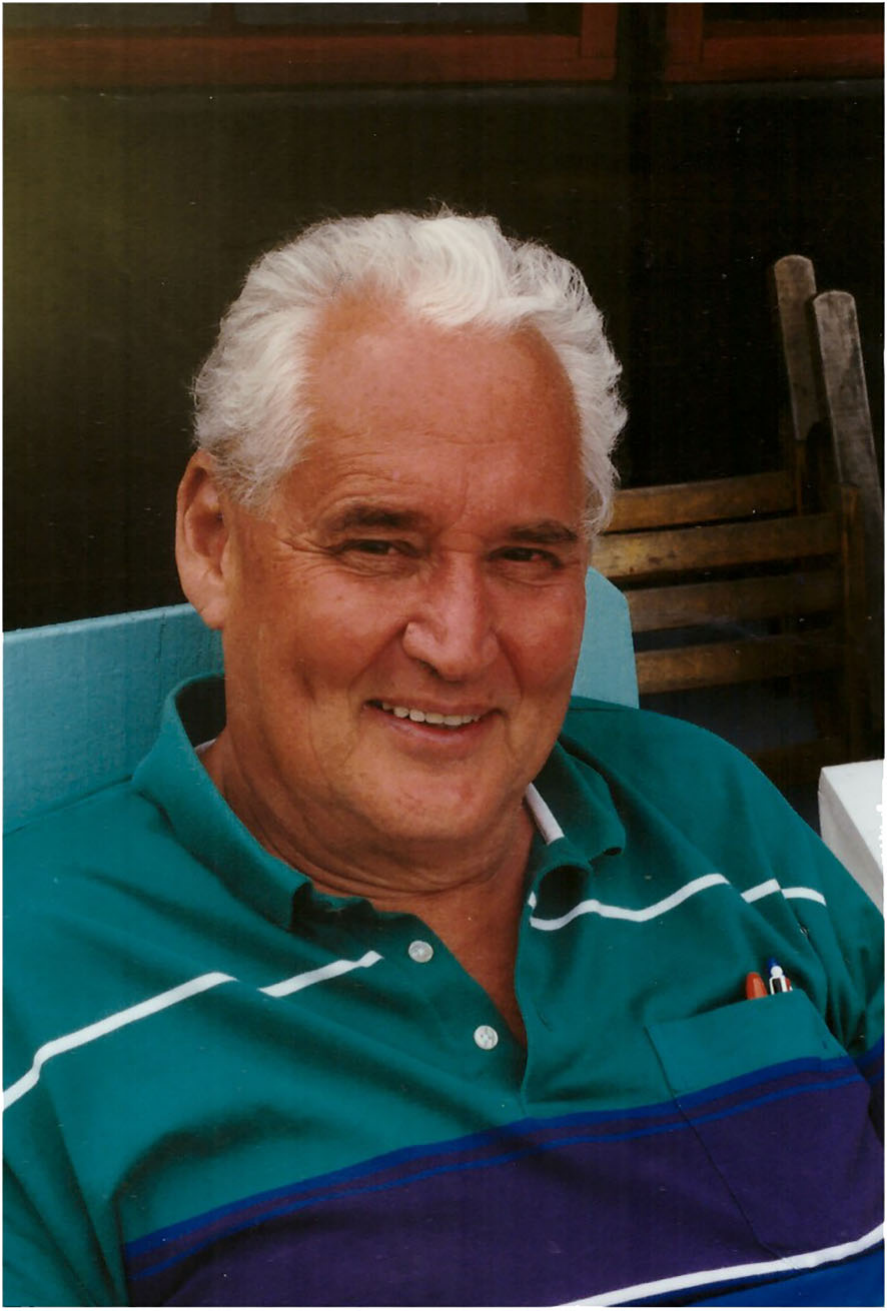
Picture of Jacques from Gilbert Vassart.

Jacques’ enthusiasm and creativity fueled a critical opinion about fashionable mainstream ideas not assessed by rigorous scientific validation, an independence that he did not hesitate to display in his academic environment, or to express vocally in discussions following presentations at scientific meetings.

His awards and honors include the Pfizer Prize (1975), the Henning (1989) and Lissitzky (2007) Prizes, the Dautrebande Prize (1992), and Francqui chairs in 1992, 1994, 1998 and 1999. He was a founding member (1966) and President (1996-1998) of the European Thyroid Association and President of the Belgian Society of Biochemistry and Molecular Biology (2002-2004). He was a member of the Belgian Royal Academy of Medicine since 1992.

Jacques is survived by his wife and co-worker Jacqueline Van Sande, his three children and six grandchildren.

## Ethics statement

Written informed consent was obtained from the individual's next of kin for the publication of any potentially identifiable images or data included in this article.

## Author contributions

All authors listed have made a substantial, direct, and intellectual contribution to the work, and approved it for publication.

